# Supplementary material for: Mechanisms governing the pioneering and redistribution capabilities of the non-classical pioneer PU.1
Source: Nat Commun. 2020 Jan 21;11:402. doi: 10.1038/s41467-019-13960-2 (PMC6972792; doi:10.1038/s41467-019-13960-2)
Supplement: Supplementary file 7 — Source data [file 41467_2019_13960_MOESM7_ESM.zip › Source_Data/FigureS2/FigureS2B_MotifScanOutput/KmeansCluster13/homerResults/motif3.similar.html]

motif3

## Information for motif3

C
T
A
G
C
T
A
G
A
T
C
G
T
G
A
C
C
T
A
G
A
T
C
G
T
C
G
A
T
C
G
A
T
A
G
C
G
A
T
C
G
C
T
A
  
Reverse Opposite:  

C
G
A
T
C
T
A
G
A
T
C
G
A
G
C
T
A
G
C
T
A
T
G
C
G
A
T
C
A
C
T
G
T
A
G
C
G
A
T
C
G
A
T
C
  

|  |  |
| --- | --- |
| p-value: | 1e-65 |
| log p-value: | -1.507e+02 |
| Information Content per bp: | 1.602 |
| Number of Target Sequences with motif | 537.0 |
| Percentage of Target Sequences with motif | 31.74% |
| Number of Background Sequences with motif | 6663.6 |
| Percentage of Background Sequences with motif | 14.78% |
| Average Position of motif in Targets | 99.9 +/- 53.2bp |
| Average Position of motif in Background | 100.7 +/- 67.6bp |
| Strand Bias (log2 ratio + to - strand density) | 0.0 |
| Multiplicity (# of sites on avg that occur together) | 1.18 |
| Motif File: | file (matrix) reverse opposite |

### Similar de novo motifs found

|  |  |  |  |  |  |  |  |
| --- | --- | --- | --- | --- | --- | --- | --- |
| Rank | Match Score | Redundant Motif | P-value | log P-value | % of Targets | % of Background | Motif file |
| 1 | 0.816 | C A T G A G T C G A T C A G T C A G T C C T A G A G T C A G T C A G T C G T A C | 1e-41 | -96.360719 | 36.94% | 22.00% | motif file (matrix) |
| 2 | 0.760 | G A T C A G T C A G T C C T A G A G T C A G T C A G T C | 1e-24 | -56.808619 | 29.85% | 19.12% | motif file (matrix) |
| 3 | 0.807 | G T C A G C A T C A T G A C T G A T G C G A T C T G A C G T A C C T A G A T G C G T A C G A T C | 1e-23 | -54.804656 | 21.93% | 12.77% | motif file (matrix) |
| 4 | 0.785 | A T C G A G C T G A C T A G T C A G T C C A T G A G T C A G T C A G T C A G T C A G T C C G A T A T G C | 1e-22 | -50.905822 | 4.61% | 1.14% | motif file (matrix) |
| 5 | 0.754 | A C T G A C T G A C T G G T C A C T A G A C T G C G T A A C T G | 1e-19 | -43.940965 | 21.45% | 13.26% | motif file (matrix) |
